# Supplementary material for: Treatment outcomes among children and adolescents with extensively drug–resistant (XDR) and pre–XDR tuberculosis: Systematic review and meta–analysis
Source: PLOS Glob Public Health. 2025 Jan 29;5(1):e0003754. doi: 10.1371/journal.pgph.0003754 (PMC11778756; doi:10.1371/journal.pgph.0003754)
Supplement: S1 Table — Note: †Data represents all XDR cases of all ages as the information was not specified for pediatric cases only. PTB: Pulmonary tuberculosis, EPTB: Extrapulmonary tuberculosis, NS: Not specified. (PDF) [file pgph.0003754.s001.pdf]

S1 Table: Additional clinical characteristics of XDR TB patients

Study	Type of TB	Previously treated (Y/N, %)	Previous contact with an MDR or XDR TB case (Y/N, %)	Clinical/radiologic presentation
**Population–based**				
Hicks et al (2014)	PTB (72.6%)<br>PTB and EPTB (27.4%)	NS	NS	NS
Kuksa et al (2014)†	PTB (93%)<br>EPTB (1%)<br>PTB and EPTB (6%)<br>XDR: PTB (94%), EPTB (3%), PTB and EPTB (3%)	Y (20%)	NS	NS
Mignone et al (2014)	PTB (72.8%)<br>EPTB (9%)<br>PTB and EPTB (18.2%)<br>XDR: PTB (50%), PTB and EPTB (50%)	N	NS	Fever, vomiting, chest pain
Seddon et al (2014)	PTB (80.5%)<br>EPTB (8.1%)<br>PTB and EPTB (11.4%)	NS	Y (50%)	Perihilar infiltrates, Hilar lymphadenopathy, Lobar/segmental collapse or opacification, Pleural effusion, Cavities
Isaakidis et al (2013)	PTB (46%)<br>EPTB (18%)<br>PTB and EPTB (36%)	Y (91%)	NS	NS
Gegia et al (2013)	PTB (25.0%)<br>EPTB (70.5%)<br>PTB and EPTB (4.5%)<br>XDR: PTB (100%)	Y (52.3%)	Y (100%)	PTB: unilateral lesions on left/right upper lobe (100%). EPTB: Peripheral lymphadenopathy (74.2%), TB meningitis (12.9%) and TB pleurisy (12.9%)
Williams et al (2013)	PTB (70.6%)<br>EPTB (17.6%)<br>PTB and EPTB (11.8%)<br>XDR: PTB (67%), EPTB (33%)	Y (100%)	NS	Cough, fever, weight loss, night sweats, lethargy
Rose et al (2012)	PTB (100%)	Y (100%)	N	Pneumonia, bronchial compression, perihilar/paratracheal lymphadenopathy, cavitation, extensive bronchiectasis
Seddon et al (2012)	PTB only (65.8%)<br>EPTB only (10.8%)<br>PTB and EPTB (23.4%)	Y (25.2%)	Y(40.5%)	Hilar lymphadenopathy or airway compression, Lobar/segmental collapse or opacification, Large pleural effusion, Cavities, Miliary opacification, Widespread bronchopneumonic changes
Liu et al (2011)†	PTB (92.2%)<br>EPTB (7.8%)<br>XDR: PTB (93.8%), EPTB (6.2%)	Y (68.8%)	NS	NS
Thomas et al (2010)	PTB (100%)	N	Y (25%)	Cavitary disease and extensive infiltrates, Perihilar lymphadenopathy
**Case–studies**				
Salazar–Austin et al 2015	PTB	Y	N	Fever, tachycardia, tachnpnoea, low body weight, left lower lobe infiltrate and hilar adenopathy
Alsleben et al (2014)	EPTB (TB meningitis)	Y	Y	Fever, abdominal discomfort, worsening headache, periorbital edema,vomiting, ataxia, meningeal enhancement
Mohan et al (2014)	EPTB (spinal)	Y	NS	Spinal abscess, cord compression, paraparesis, kyphotic deformity in the upper back
Rodrigues et al (2014)	PTB	N	N	Vespertine fever, chest pain, nonproductive cough, anorexia, consolidation in the upper left lobe, endobronchic dissemination and calcified mediastinic adenopathies
Uppuluri et al (2014)	PTB	Y	N	Cough, fever, weight loss, left lower lobe consolidation
Katragkou et al (2013)	PTB	Y	N	Fever, cought, weight loss
	EPTB (Central Nervous System TB)	N	Y	Fever, cough, vomit, and tonic–clonic seizures
Payen et al (2012)	PTB	Y	NS	NS
Dauby et al (2011)	PTB	Y	N	Fever, cough, anorexia, failure to thrive, lesion, severely damaged left lung
Kjollerstrom et al (2011)	PTB (100%)	Y	Y	Hemoptysis (blood in sputum from lungs)
	EPTB (spinal)	Y	N	Cough & fever lasting 1 mo, left foot swelling and discharge (non-foul smelling), weight loss
Shah et al (2011)	PTB	N	N	Cough, fever, consolidation left upper lobe, bilateral inguinal adenopathy with left sided otorrhea, hepatomegaly
	EPTB (abdominal)	Y	Y	Dry cough, fever and abdominal pain lasting 1 mo, reduced appetite, difficult to gain weight, non significant cervical, inguinal and axillary lymph nodes, hepatomegaly
Anger et al (2010)	PTB and EPTB (pericardial, peritoneal)	Y	NS	NS
Kulkarni et al 2009	EPTB (meningitis, lymphadenopathy)	Y	N	Fever, headache, lethargy, loss of appetite, upper abdominal pain
Schaaf et al. 2009	PTB	Y	Y	Cough, fever, consolidation in the left upper lobe, bilateral inguinal adenopathy with left sided otorrhea, and hepatomegaly
		Y	N	Fever, failure to thrive, lymphadenopathy
Schulger et al 1996	PTB (100%)	N	N	Cough, fever, low body weight/height (10th percentile), infiltrates/calcification in the right lobe

Note: †Data represent all XDR cases of all ages as the information was not specified for pediatric cases only. PTB: Pulmonary tuberculosis, EPTB: Extrapulmonary tuberculosis, NS: Not specified.
