# Supplementary material for: Treatment outcomes among children and adolescents with extensively drug–resistant (XDR) and pre–XDR tuberculosis: Systematic review and meta–analysis
Source: PLOS Glob Public Health. 2025 Jan 29;5(1):e0003754. doi: 10.1371/journal.pgph.0003754 (PMC11778756; doi:10.1371/journal.pgph.0003754)
Supplement: S2 Table — Note: (A) Quality assessment of studies included in the meta-analysis. (B) Quality assessment of studies excluded in the meta-analysis due to missing data on key outcomes such as adverse events and treatment regimens, and data points required to calculate pooled proportions. These studies were included to provide a comprehensive review of current literature. ‡ Long term follow-up is classified as any treatment duration > 12 months; NS: Not specified; NA: Not available. (PDF) [file pgph.0003754.s002.pdf]

S2 Table: Quality assessment using Newcastle-Ottawa Scale

	Quality assessment using Newcastle-Ottawa Scale								
	Selection				Measurement		Outcome		
**Study**	Representativeness of the cohort to the average patient on XDR/pre-XDR treatment†	Ascertainment of XDR/pre-XDR	Baseline XDR/pre-XDR resistance testing done	Risk of bias (high, medium, low)	XDR/pre-XDR confirmed through DST	Risk of bias	Average follow-up of 1 year post-treatment initiation	Long-term follow-up of <30% among patients onXDR/pre-XDR treatment‡	Risk of bias
**Population-based studies**									
Hicks et al (2014)	-	*	*	medium	*	low-medium	*	-	medium
Kuksa et al (2014)	*	*	*	low	*	low-medium	*	-	medium
Mignone et al (2014)	*	*	*	low	*	low-medium	*	-	medium
Seddon et al (2014)	*	*	*	low	*	low-medium	*	*	medium
Isaakidis et al (2013)	-	*	*	medium	*	low-medium	-	-	high
Gegia et al (2013)	*	*	*	low	*	low-medium	*	-	medium
Williams et al (2013)	*	*	*	low	*	low-medium	*	-	medium
Rose et al (2012)	*	*	*	low	*	low-medium	*	-	medium
Seddon et al (2012)	*	*	*	low	*	low-medium	*		medium
Liu et al (2011)	*	*	*	low	*	low-medium	*	-	medium
Thomas et al (2010)	*	*	*	low	*	low-medium	*	-	medium
**Case studies**									
Salazar-Austin et al (2015)	-	*	*	medium	*	low-medium	*	-	medium
Alsleben et al (2014)	-	*	*	medium	*	low-medium	*	-	medium
Mohan et al (2014)	-	*	*	medium	*	low-medium	-	*	medium
Rodrigues et al (2014)	*	*	*	low	*	low-medium	*	-	medium
Uppuluri et al (2014)	-	*	*	medium	*	low-medium	*	*	low
Katragkou et al (2013)	*	*	*	low	*	low-medium	-	*	medium
Payen et al (2012)	-	*	*	medium	*	low-medium	*	-	medium
Dauby et al (2011)	-	*	*	medium	*	low-medium	*	-	medium
Kjollerstrom et al (2011)	*	*	*	low	*	low-medium	*	-	medium
Shah et al (2011)	*	*	*	low	*	low-medium	*	-	medium
Anger et al (2010)	-	*	*	medium	*	low-medium	*	-	medium
Kulkarni et al (2009)	-	*	*	medium	*	low-medium	NS	NS	NA
Schaaf et al (2009)	-	*	*	medium	*	low-medium	*	-	medium
Schulger et al (1996)	*	*	*	low	*	low-medium	*	*	low

Note: (A) Quality assessment of studies included in the meta-analysis. (B) Quality assessment of studies excluded in the meta-analysis due to missing data on key outcomes such as adverse events and treatment regimens, and data points required to calculate pooled proportions. These studies were included to provide a comprehensive review of current literature. † Case studies/series were awarded one star if the sample of XDR/pre-XDR treatment patients was >1; ‡ Long term follow-up is classified as any treatment duration >12 months; NS: Not specified; NA: Not available.

S2 Table: Quality assessment using Newcastle-Ottawa Scale

Study	Selection: Representativeness of the cohort to the average patient on XDR/pre-XDR treatment[†]	Selection: Ascertainment of XDR/pre-XDR	Selection: Baseline XDR/pre-XDR resistance testing done	Selection: Risk of bias (high, medium, low)	Measurement: XDR/pre-XDR confirmed through DST	Measurement: Risk of bias	Outcome: Average follow-up of 1 year post-treatment initiation	Outcome: Long-term follow-up of <30% among patients on XDR/pre-XDR treatment[‡]	Outcome: Risk of bias
**Population-based studies**									
Malik et al (2022)	-	*	*	medium	*	low-medium	*	-	medium
Shetty et al (2022)	*	*	*	low	*	low-medium	*	*	low
Desai et al (2019)	*	*	*	low	*	low-medium	*	-	medium
Madzgharashvili et al (2021)	*	*	*	low	*	low-medium	*	-	medium
Tola et al (2020)	-	*	*	medium	*	low-medium	*	-	medium
Pinto et al (2021)	*	*	-	medium	*	low-medium	*	-	medium
Dhakulkar et al (2021)	*	*	*	low	*	low-medium	*	*	low
Abubakar et al (2022)	*	*	-	medium	*	low-medium	*	-	medium
Smirnova et al (2016)	*	*	*	low	*	low-medium	*	-	medium
Moore et al (2015)	*	-	-	high	-	medium-high	*	-	medium
Seddon et al (2012)	*	*	-	medium	*	low-medium	*	-	medium
Seddon et al (2014)	-	*	-	high	*	low-medium	*	-	medium
Vukugah et al (2019)	-	*	*	medium	-	medium-high	*	-	medium
Naz et al (2021)	-	*	*	medium	-	medium-high	-	*	medium
Pirmahmadzoda et al (2021)	*	*	-	medium	*	low-medium	*	-	medium
Schaaf et al (2020)	-	*	-	high	*	low-medium	NS	-	medium-high
Kalawadia et al (2024)	*	*	-	medium	*	low-medium	NS	-	medium-high
Sharma et al (2020)	-	*	-	high	*	low-medium	*	-	medium
Das et al (2020)	-	*	*	medium	*	low-medium	*	-	medium
Jantarabenjakul et al (2022)	*	*	*	low	*	low-medium	*	-	medium
Sun et al (2023)	*	*	*	low	*	low-medium	*	NS	low-medium
Schafer et al (2023)	-	*	*	medium	*	low-medium	*	-	medium
Khantee et al (2021)	-	*	*	medium	-	medium-high	*	-	medium

Note: (A) Quality assessment of studies included in the meta-analysis. (B) Quality assessment of studies excluded in the meta-analysis due to missing data on key outcomes such as adverse events and treatment regimens, and data points required to calculate pooled proportions. These studies were included to provide a comprehensive review of current literature. † Case studies/series were awarded one star if the sample of XDR/pre-XDR treatment patients was >1; ‡ Long term follow-up is classified as any treatment duration >12 months; NS: Not specified; NA: Not available.

NEWCASTLE – OTTAWA QUALITY ASSESSMENT SCALE (MODIFIED)

Note: A study can be awarded a maximum of one star for each numbered item within the Selection, Measurement and Outcome categories.

Selection

1) Representativeness of the exposed cases:

   a) Truly representative of the average patient on XDR treatment in the community. □
   b) Somewhat representative of the average patient on XDR treatment in the community. □
   c) Selected group of users.
   d) No description of the sampling strategy.

2) Ascertainment of XDR/pre–XDR:
   a) Secure record (e.g. medical records). □
   b) Structured interview.
   c) Written self–report.
   d) No description.

3) Resistance testing:
   a) Baseline XDR/pre–XDR resistance testing done to isoniazid and rifampin, plus any fluroquinolone and at least one of three injectable second–line drugs.□
   b) No description of resistance testing done.

Measurement

1) Ascertainment of XDR:
   a) Validated assessment (e.g. DST).□
   b) Self–report.
   c) No description.

Outcome

1) Was follow–up long enough for outcomes to occur?
   a) Yes, average follow–up of at least one year post–treatment initiation. □
   b) No, average follow–up less than one year post–treatment initiation.
   c) No description.

2) Long–term follow–up of greater than one year in less than 30% of patients on XDR/pre–XDR treatment:
   a) Yes.□
   b) No.

This scale has been adapted from the Newcastle–Ottawa Quality Assessment Scale (NOS) to perform a quality assessment of population–based (cohort) and case studies for the systematic review "Treatment outcomes among children and adolescents with extensively drug–resistant (XDR) and pre–XDR tuberculosis : systematic review and meta–analysis." This scale is a modified version of the NOS scale, previously used by Dey et al. (2013), in which they adapted it to properly assess the quality of randomized trials, observational studies and case studies.

We did a comprehensive search on literature and used the following revised scoring algorithm based on literature (see McPheeters et al. 2012; see Appendix G page 103–104 in http://www.ncbi.nlm.nih.gov/pubmedhealth/PMH0049229/).

Scoring algorithm

**Quality rating**	**# Points in Selection Domain**	**# Points in Measurement Domain**	**# Points in Outcome Domain**
Good/ low risk of bias (RoB)	3	1	2
Fair/ medium RoB	2	1	1
Poor/ high RoB	0–1	0	0

References

Wells G, Shea B, O'Connell D, et al. The Newcastle–Ottawa Scale (NOS) for assessing the quality of nonrandomized studies in meta–analyses. *Proceedings of the 3rd Symposium on Systematic Reviews*. *Beyond the basics: improving quality and impact*; 2000 July 3–5; Oxford. Oxford, 2000.

Dey T, Brigden G, Cox H, Shubber Z, Cooke G, Ford N. Outcomes of clofazimine for the treatment of drug–resistant tuberculosis: a systematic review and meta–analysis. *J Antimicrob Chemother* 2012; **68**:284–93.

McPheeters ML, Kripalini S, Peterson NB, Idowu RT, Jerome RN, Potter SA, et al. Quality improvement interventions to address health disparities. Closing the Quality Gap: Revisiting the State of Science [Internet]. Rockville (MD): Agency for Healthcare Research and Quality (US); 2000 [updated 2007 Aug; cited 2016 Mar 20]. Available from: http://www.ncbi.nlm.nih.gov/pubmedhealth/PMH0049222/pdf/TOC.pdf
