# Supplementary material for: Treatment outcomes among children and adolescents with extensively drug–resistant (XDR) and pre–XDR tuberculosis: Systematic review and meta–analysis
Source: PLOS Glob Public Health. 2025 Jan 29;5(1):e0003754. doi: 10.1371/journal.pgph.0003754 (PMC11778756; doi:10.1371/journal.pgph.0003754)
Supplement: S3 Table — *Represents the overall sample, including MDR/DRTB cases; information for XDR cases is shown if specified in the study. †The majority of XDR cases were culture–positive and were treated based on their DST results except for the cases in Seddon et al (2014), where 50% of the XDR cases were culture–confirmed, and thus, DST was completed on 50% of cases’ samples; the remaining 50% cases were treated based on the DST results from the source. ‡HIV status was not known (Williams et al (2013)) or tested (Gegia et al (2013)) for all XDR cases. XDR: Extensively drug resistant, P cohort: Prospective cohort, R cohort: Retrospective cohort, NS: Not specified, DST: Drug susceptibility test, TST: Tuberculosis Skin Test, CXR: Chest X–Ray, CT scan: Computerized Tomography. (PDF) [file pgph.0003754.s003.pdf]

S3 Table: Description of included studies and demographic characteristics of patients

Among Total Sample*									Among XDR Cases Only	
**Study**	**Location**	**National TB prevalence[1] (per 10^5)**	**Study period**	**(a) Study design<br>(b) Study setting**	**Median age (years, range)**	**Gender (%)**	**Sample size**	**Number of cases (XDR/pre-XDR)**	**(a) Method of diagnosis<br>(b) Clinical/Radiologic presentation**	**(a) HIV prevalence (%)<br>(b) Other co-morbid conditions (%)**
**Population-based**										
Malik et al (2022)	Pakistan	220	2014-2019	(a) R Cohort<br>(b) Facility	16 (13-18)	M (32.4%)<br>F (67.6%)	247	46 (3 XDR, 43 pre–XDR)	(a) Culture test, DST, LPA, clinical presentation<br>(b) parenchymal infiltrates, cavitation	(a) 33%<br>(b) NS
Shetty et al (2022)	India	195	2013-2018	(a) R Cohort<br>(b) Facility	7 (0.1-17)	M (50.0%)<br>F (50.0%)	174	56 (9 XDR, 47 pre-XDR)	(a) Culture test (using 2 sputum samples), DST, LPA, GeneXpert<br>(b) Blurry vision, ophthalmalgia, optic neuritis, neutropenia, anemia, eosinophilia	(a) 11%<br>(b) Lissencephaly
Desai et al (2019)	India	195	2012-2014	(a) R Cohort<br>(b) Facility	..	M (30.4%)<br>F (69.6%)	1743	26 (3 XDR, 23 pre-XDR)	(a) Culture test, DST, LPA, clinical presentation<br>(b) NS	(a) None<br>(b) Anemia, diabetes mellitus, hypothyroidism, depression, nonseminomatous germ cell tumor of testis, renal failure, elephantiasis and mental retardation, hypertension, and urolithiasis
Madzgharashvili et al (2021)	Georgia	2	2009-2016	(a) R Cohort<br>(b) National	14 (4.6-16.0)	M (60.5%)<br>F (39.5%)	124	35 (12 XDR, 23 pre-XDR)	(a) Culture test, DST<br>(b) Nausea, joint pain/arthralgia, anxiety, gastrointestinal tract disturbance, rash	(a) None<br>(b) NS
Tola et al (2020)	Ethiopia	119	2009-2019	(a) R Cohort<br>(b) National	..	M (43.2%)<br>F (56.8%)	155	..	(a) Culture test, DST, LPA, GeneXpert, clinical presentation<br>(b) NS	(a) 9%<br>(b) NS
Pinto et al (2021)	India	195	2014-2017	(a) R Cohort<br>(b) Facility	5 (2-9)	M (48.8%)<br>F (51.2%)	41	..	(a) Culture test, DST, GeneXpert<br>(b) Pain, neurological deficit, cold abscess and deformity; radiographic findings of vertebral collapse, kyphosis, soft tissue shadow — suggestive of cold abscess	(a) NS<br>(b) NS
Dhakulkar et al (2021)	India	195	2017-2018	(a) R Cohort<br>(b) Facility	17 (4-19)	M (28.4%)<br>F (71.6%)	268	113 (35 XDR, 78 pre-XDR)	(a) Culture test, DST, LPA, GeneXpert<br>(b) NS	(a) 2.9%<br>(b) NS
Abubakar et al (2022)	Pakistan	220	2010-2019	(a) R Cohort<br>(b) National	7 (0.1-14)	M (58.3%)<br>F (46.7%)	42	11	(a) Culture test, DST<br>(b) NS	(a) NS<br>(b) None
Smirnova et al (2016)	Northwest Russia	31	2001-2012	(a) R Cohort<br>(b) National	11 (5.0–16)	M (40.4%)<br>F (59.6%)	52	..	(a) Culture test, DST, molecular testing<br>(b) Cough, fever, weight loss	(a) None<br>(b) NS
Moore et al (2015)	South Africa	696	2005-2010	(a) R Cohort<br>(b) National	11 (0.1-18)	M (44.1%)<br>F (55.9%)	774	36	(a) NS<br>(b) Cough, fever, night sweats, weight loss	(a) 54.7%<br>(b) NS
Seddon et al (2012)	South Africa	696	2003-2008	(a) R Cohort<br>(b) Facility	4 (2-9)	M (41.4%)<br>F (58.6%)	111	5	(a) Culture test, DST<br>(b) Radiological evidence of intrathoracic disease.	(a) 43%<br>(b) NS
Seddon et al (2014)	South Africa	696	2009-2010	(a) Combined R & P Cohort<br>(b) Facility	3 (1.5-5.5)	M (46.3%)<br>F (53.7%)	149	6	(a) Culture test, DST<br>(b) Radiological evidence of intrathoracic TB.	(a) 33.3%<br>(b) NS
Vukugah et al (2019)	Central Africa	540	2018-2020	(a) R Cohort<br>(b) Facility	6 (2-12)	M (49.7%)<br>F (50.3%)	610	..	(a) Smear and culture test, CXR, GeneXpert<br>(b) NS	(a) 25.1%<br>(b) NS
Naz et al (2021)	Pakistan	220	2010-2019	(a) R Cohort<br>(b) Facility (multicentre)	12 (10-14)	M (26%)<br>F (74%)	213	48 (pre-XDR)	(a) Smear and culture test, GeneXpert<br>(b) NS	(a) NS<br>(b) Diabetes mellitus, depression, epilepsy
Pirmahmadzoda et al (2021)	Tajikistan	88	2013-2019	(a) R Cohort<br>(b) National	10 (0.1-17)	M (33.3%)<br>F (66.7%)	60	19 (8 XDR, 11 Pre-XDR)	(a) Smear and culture test, DST, GeneXpert<br>(b) NS	(a) 1.6%<br>(b) NS
Schaaf et al (2020)	South Africa	696	2013-2017	(a) P Cohort<br>(b) Facility	3 (1.17-6.58)	M (51.8%)<br>F (48.2%)	587	2 (1 XDR, 1 pre-XDR)	(a) Smear and culture test, DST, GeneXpert<br>(b) NS	(a) 12.9%<br>(b) NS
Kalawadia et al (2024)	India	195	2018-2021	(a) R Cohort<br>(b) Facility	13 (6-17)	M (21.7%)<br>F (78.3%)	60	50 (27 XDR, 23 pre-XDR)	(a) Culture test, DST,<br>(b) NS	(a) NS<br>(b) NS

Sharma et al (2020)	India	195	2013-2019	(a) R Cohort<br>(b) National	11 (0.1-14)	M (21.6%)<br>F (78.4%)	1380	9 (4 XDR, 5 pre-XDR)	(a) Culture test, DST, LPA,<br>(b) NS	(a) 2.7%<br>(b) NS
Das et al (2020)	India	195	2014-2020	(a) R Cohort<br>(b) Facility	15.5 (3-19)	M (37%)<br>F (63%)	24	24 (16 XDR, 8 pre-XDR)	(a) Culture test, DST, GeneXpert<br>(b) NS	(a) None<br>(b) NS
Jantarabenjakul et al (2022)	Thailand	150	2006-2021	(a) R Cohort<br>(b) Facility	12.2 (7.3-14.2)	M (44%)<br>F (56%)	163	8 (pre-XDR)	(a) Culture test, DST<br>(b) NS	(a) 17%<br>(b) NS
Sun et al.	China	89	2017-2021	(a) R Cohort	15 (0.1-18)	M (53.0%)<br>F (47.0%)	2976	28 (pre-XDR)	(a) Culture test, DST, GeneXpert<br>(b) NS	(a) NS<br>(b) NS
Schäfer et al (2023)	Germany	5	2010-2020	(a) R Cohort<br>(b) Facility	..	M (54.2%)<br>F (45.8%)	52	52	(a) Culture test, DST, GeneXpert<br>(b) NS	(a) NS<br>(b) NS
Khantee et al (2021)	Thailand	150	2007-2018	(a) R Cohort<br>(b) Facility	5.5 (1.7–12.8)	M (45.8%)<br>F (54.2%)	177	1	(a) Culture test<br>(b) Fever, cough, dyspnea, hemoptysis, lymphadenopathy, weight loss, hepatomegaly, infiltrates/calcification	(a) 38.9%<br>(b) NS

Note: The data above is for studies excluded from the meta-analysis, but included to ensure extensive survey of current literature. *Represents the overall sample, including MDR/DRTB cases; information for XDR cases is shown if specified in the study. †The majority of XDR cases were culture–positive and were treated based on their DST results except for the cases in Seddon et al (2014), where 50% of the XDR cases were culture–confirmed, and thus, DST was completed on 50% of cases' samples; the remaining 50% cases were treated based on the DST results from the source. ‡HIV status was not known (Williams et al (2013)) or tested (Gegia et al (2013)) for all XDR cases. XDR: Extensively drug resistant, P cohort: prospective cohort, R cohort: retrospective cohort, NS: Not specified, DST: Drug sensitivity test, TST: Tuberculosis Skin Test, CXR: Chest X–Ray, CT scan: Computerized Tomography.
