# Supplementary material for: Treatment outcomes among children and adolescents with extensively drug–resistant (XDR) and pre–XDR tuberculosis: Systematic review and meta–analysis
Source: PLOS Glob Public Health. 2025 Jan 29;5(1):e0003754. doi: 10.1371/journal.pgph.0003754 (PMC11778756; doi:10.1371/journal.pgph.0003754)
Supplement: S4 Table — Note: The data above is for studies excluded from the meta-analysis, but included to ensure extensive survey of current literature. *Represents median or average length of treatment for population–based studies, and the total length of treatment for case–based studies. †Patient remains on treatment, including only case 2 from Shah et al (2011). DST: Drug susceptibility testing, CSF: Cerebrospinal fluid, Tx: Treatment, NS: Not specified, NA: Not applicable, FQ: Fluoroquinolone, H: Isoniazid, R: Rifampicin, E: Ethambutol, Z: Pyrazinamide, Rfb: Rifabutin, Km: Kanamycin, Amk: Amikacin, Cm: Capreomycin, S: Streptomycin, Lfx: Levofloxacin, Mfx: Moxifloxacin, Ofx: Ofloxacin, Cfx: Ciprofloxacin, Gfx: Gatifloxacin, Eto: Ethionamide, Pto: Protionamide, Cs: Cycloserine, Trd: Terizidone, PAS: p–aminosalicylic acid, Cfz: Clofazimine, Lzd: Linezolid, Amx/Clv: Amoxicillin/clavulanate, Thz: Thioacetazone, Clr: Clarithromycin Clr, Ipm: Imipenem, High–dose H: High dose isoniazid, Th: Thiacetazone, Rpt: Rifapentine, Pto: Prothionamide, Rif: Rifampin, Mem: Meropenem, M–C: Meropenem–Clavulanate, Ipm/Cln: Imipenem/cilastatin. (PDF) [file pgph.0003754.s004.pdf]

S4 Table: Description of treatment regimen

Study	Length of Treatment (months)*	Treatment Description	Injectables Used	Fluoroquinolones used	Resistance Pattern	Definition of Success
**Population-based Studies**						
Malik et al (2022)	20	Individualized based on DST results. For patients without resistance to SLD, treatment consisted of ≥ 8 months with a combination of Amk, Km, Cm, Lfx, Eto, Cs, and Z, followed by 12 months with Lfx, Eto, Cs, and Z. Patients resistant to SLD received PAS in addition to the regimen for ≥ 20 months.	Am or Km or Cm	Lfx	H, R, at least 1 injectable (Am/Km/Cm) and 1 FQ	Cure or tx completion (WHO)
Shetty et al (2022)	18*	Individualized based on DST results. Drugs used: One injectable (Amk preferred over Km, Cm), R, E, and others not specified.	NS	Mfx	H, R, Z, E, S, Eto, Am, Km, Cm, Ofx, Mfx, PAS, Cfz	tx completion
Desai et al (2019)	24	Individualized based on DST results. Drugs used: One injectable (Am, Km, or Cm), R, H, AM, FQ.	Cm	NS	NS	Cure or tx completion (WHO)
Madzgharashvili et al (2021)	20*	Individualized based on DST results. Drugs used: One injectable (Amk, Km, or Cm), R, H, E, Lzd, Dlm, BDQ.	NS	NS	H, R, Z, E, Km, Cm, Ofx, Pto, PAS	Cure or tx completion (WHO)
Tola et al (2020)	20	Individualized based on DST results. Drugs used: One injectable (Amk, Km, or Cm), R, E, Lfx, Eto, Cs, PAS, Z, Pto, Lzd, Cfz.	NS	NS	NS	Cure or tx completion (WHO)
Pinto et al (2021)	12	Individualized based on DST results. Drugs used: One injectable (Amk, Km, or Cm), R, E, R, H, Z, E, Mfx, Cs, Cfz, PAS.	One of Amk/Mfx/Cs/Cfz/PAS	NS	H, R, Z, E	tx completion (WHO) and partial or complete clinical and radiological resolution of symptoms
Dhakulkar et al (2021)	24*	Individualized based on DST results. Drugs used: One injectable (Amk, Km, or Cm), R, E, and others not specified.	NS	NS	NS	Cure or tx completion (WHO)
Abubakar et al (2022)	25*	Individualized based on DST results. Drugs used: One injectable (Amk, Km, or Cm), R, E, PAS, Z, Cs, Eto, Lzd, Mfx, Cm, Cfz, Lfx, Clr, High–dose H, and BDQ.	Amk or Km or Cm	Lfx or Mfx	H, Z, E, S, Km, Cm, Amk, Lfx, Eto, Mfx, Lzd, Clr	Cure or tx completion (WHO)
Smirnova et al (2016)	22	Based on existing WHO recommendations. Drugs used: One injectable (Amk, Km, or Cm), R, E, and others not specified	NS	NS	NS	Cure or tx completion (WHO)
Moore et al (2015)	22	Individualized based on DST results. Drugs used: One injectable (Amk, Km or Cm) R, E, others not specified.	Amk or Km or Cm	Cfx or Ofx or Mfx	H, R, Z, E, S, Km, Cm, Eto	Cure or tx completion (WHO)
Seddon et al (2012)	18	Individualized based on DST results. Drugs used: One injectable (Amk, Km or Cm) R, E, others not specified.	Amk or Cm	Ofx	H, R, Z, E, S, Amk, Cm, Ofx, Eto, Cs, PAS, Clr, Amx/Clv	Cure (3 consecutive negative respiratory cultures obtained at least 1 month apart with no positive cultures from the date of the first negative result) and tx completion
Seddon et al (2014)	13	Individualized based on DST results. Drugs used: One injectable (Amk, Km or Cm) R, E, others not specified.	NS	NS	H, R, at least 1 injectable and/or 1 FQ	Clinical improvement or negative cultures at tx completion
Vukugah et al (2019)	24	Based on existing WHO recommendations. Drugs used: One injectable (Amk, Km or Cm) R, E, others not specified.	NS	NS	NS	Cure (negative-smear in the last month of treatment and on at least 1 previous occasion 30 days apart) or tx completion
Naz et al (2021)	20	Individualized based on DST results. Drugs used: One injectable (Amk, Km, or Cm), Lfx, Eto, Cs, Z.	Amk or Km or Cm	NS	H, R, Z, E, S, Eto	Cure or tx completion (WHO)
Pirmahmadzoda et al (2021)	24*	Individualized based on DST results. Drugs used: One injectable (Amk, Km, or Cm), Lfx, Eto, Cs.	Amk or Cm	Lfx or Mfx	H, R, Z, E, S, Km, Cm, Am Lfx, Mfx, Pto	Cure or tx completion (WHO)
Schaaf et al (2020)	24	Individualized based on DST results. Drugs used: One injectable (Amk, Km, or Cm), R, E, others not specified.	Amk	Lfx or Ofx	H, R, at least 1 injectable and 1 FQ	tx completion
Kalawadia et al (2024)	6	Individualized based on DST results. Drugs used: One injectable (Amk, Km, or Cm), R, E, others not specified.	NS	NS	NS	Cure or tx completion (WHO)
Sharma et al (2020)	24	Individualized based on DST results. Drugs used: One injectable (Amk, Km, or Cm), R, E, others not specified.	NS	NS	H, R, at least 1 injectable and/or 1 FQ	tx completion
Das et al (2020)	18	Individualized based on DST results. Drugs used: One injectable (Amk, Km, or Cm), R, E, others not specified.	Cm	Mfx	H, R, at least 1 injectable and/or 1 FQ	Cure or tx completion (WHO)
Jantarabenjakul et al (2022)	10*	Individualized based on DST results. Drugs used: One injectable (Amk, Km, or Cm), R, E, others not specified.	Amk or Km or Cm	Lfx or Ofx	H, R, at least 1 injectable and/or 1 FQ	Cure or tx completion with improved clinical/radiological status
Sun et al (2023)	24*	Individualized based on DST results.Drugs used: One injectable (Amk, Km, or Cm), R, E, others not specified.	Amk	Lfx	H, E, S, at least 1 injectable and/or 1 FQ	NS
Schäfer et al (2023)	11*	Individualized based on DST results. Drugs used: One injectable (Amk, Km or Cm) R, E, others not specified.	Amk	Lfx or Mfx	H, E, Mfx, Cs, Lzd, Ofx, Eto	Cure (tx completion, improved clinical/radiological status, and sputum culture conversion)
Khantee et al (2021)	12	Based on diagnosis and severity of tuberculosis. Drugs used: One injectable (Amk, Km or Cm) R, E, others not specified.	NS	Ofx	H, R, S, Ofx	Cure or tx completion (WHO)

Note: The data above is for studies excluded from the meta-analysis, but included to ensure extensive survey of current literature. *Represents median or average length of treatment for population–based studies, and the total length of treatment for case–based studies. †Patient remains on treatment, including only case 2 from Shah et al (2011). DST: Drug sensitivity testing, CSF: Cerebrospinal fluid, Tx: Treatment, NS: Not specified, NA: not applicable, PA: Patient, FQ: fluoroquinolone, SLD: second-line drugs, H: isoniazid, R: rifampicin, E: ethambutol, Z: Pyrazinamide, Dlm: delamanid, BDQ: bedaquiline, Rfb: rifabutin, Km: kanamycin, Amk: amikacin, Cm: capreomycin, S: streptomycin, Lfx: levofloxacin, Mfx: moxifloxacin, Ofx: ofloxacin, Cfx: Ciprofloxacin, Gfx: Gatifloxacin, Eto: ethionamide, Pto: protionamide, Cs: cycloserine, Trd: terizidone, PAS: p–aminosalicylic acid, Cfz: clofazimine, Lzd: linezolid, Amx/Clv: amoxicillin/clavulanate, Thz: thioacetazone, Clr: clarithromycin Clr, Ipm: imipenem, High–dose H: high dose isoniazid, Th: Thiacetazone, Rpt: Rifapentine, Pto: Prothionamide, Rif: Rifampin, Mem: Meropenem, M–C: Meropenem–Clavulanate, Ipm/Cln: imipenem/cilastatin.
