# Supplementary material for: Treatment outcomes among children and adolescents with extensively drug–resistant (XDR) and pre–XDR tuberculosis: Systematic review and meta–analysis
Source: PLOS Glob Public Health. 2025 Jan 29;5(1):e0003754. doi: 10.1371/journal.pgph.0003754 (PMC11778756; doi:10.1371/journal.pgph.0003754)
Supplement: S5 Table — Note: The data above is for studies excluded from the meta-analysis, but included to ensure extensive survey of current literature. *Number and proportion of XDR cases. †Personal communication with the clinical investigator (Seddon, James. Conversation with: Jay Patra. 2015 December 08, 17.). Tx: Treatment, DST: Drug susceptibility testing, CSF: Cerebrospinal fluid, N A: Not applicable, NS: Not specified, GI: Gastrointestinal, EFV: Efavirenz, D4T: Stavudine, Ct: Colistin, Z: Pyrazinamide, Rfb: Rifabutin, Km: Kanamycin, Amk: Amikacin, Cm: Capreomycin, Cfx: Ciprofloxacin, Gfx: Gatifloxacin, Eto: Ethionamide, Pto: Protionamide, Cs: Cycloserine, Trd: Terizidone, PAS: p–aminosalicylic acid, Cfz: Clofazimine, Lzd: Linezolid. (PDF) [file pgph.0003754.s005.pdf]

S5 Table: Adverse events

Study	Adverse events (n, %)*	Grade	Implicated drugs or health conditions
**Population-based studies**			
Malik et al (2022)	NS	NA	NA
Shetty et al (2022)	Osteoarticular (25, 14.4%)<br>Neurological (15, 8.6%)<br>Lymphadenopathy (27, 15.5%)<br>Subclinical hypothyroidism (12, 6.98%)<br>Optic neuritis (4, 2.3%)<br>Neutropenia (13, 7.5%)<br>Anemia (36, 20.7%)<br>Eosinophilia (27, 15.5%)	Unclear	NS
Desai et al (2019)	Gastritis (21, 27.6%)<br>Psychiatric ADR (15, 19.7%)<br>Arthralgia (6, 7.9%)<br>Ototoxicity (3, 3.9%)<br>Hepatotoxicity (3, 3.9%)<br>Hypothyroidism (3, 3.9%)<br>Skin rash (1, 1.3%)	2 to 3	NS
Madzgharashvili et al (2021)	Hypokalemia (14, 11.8%)<br>Nausea (64, 53.9%)<br>Rashes (18, 15.1%)<br>Joint pain/arthralgia (29, 24.4%)<br>GI tract disturbance (22, 18.5%)<br>Headache (12, 10.1%)<br>Anxiety (22, 18.5%)<br>Optic neuritis (1, 0.8%)<br>Hearing loss (3, 2.5%)<br>Renal failure (1, 0.8%)<br>Peripheral neuropathy (4, 3.4%)<br>Elevated liver enzymes (4, 3.4%)<br>Anemia (1, 0.8%)<br>Itchiness (1, 0.8%)<br>Seizure (5, 4.2%)<br>Thrombocytopenia (2, 1.7%)	1 to 3	Lzd
Tola et al (2020)	NS	NA	NA
Pinto et al (2021)	NS	NA	NA
Dhakulkar et al (2021)	NS	NA	NA
Abubakar et al (2022)	NS	NA	NA
Smirnova et al (2016)	Eosinophilia (5, 9.6%)<br>Hepatitis (3, 5.8%)<br>Renal toxicity (1, 2%)<br>Nausea and/or Vomiting (5, 13%)<br>Joint pain (4, 8%)<br>Hearing loss (1, 3.8%)	3 to 4<br>Unclear<br>1<br>Unclear<br>Unclear<br>1	Pto, PAS
Moore et al (2015)	NS	NA	NA
Seddon et al (2012)	NS	NA	NA
Seddon et al (2014)	Joint, muscle, or bone pain (137, 19.7%)<br>Skin rashes (137, 19.7%)<br>Itchy skin (137, 19.7%)<br>Headache (136, 19.5%)<br>Sleep/mood problem (137, 19.7%)<br>Lethargy (137, 19.7%)<br>Visual problem (137, 19.7%)<br>Vomiting (137, 19.7%)<br>Diarrhea (137, 19.7%)	1 to 4<br>1, 2, 4<br>1, 2, 4<br>1 to 2<br>1, 2, 4<br>1, 2, 4<br>1<br>1, 2, 4<br>1, 2, 4	NS

	Jaundice (137, 19.7%)<br>Appetite/nausea (137, 19.7%)<br>Hearing loss (142, 20.4%)	1, 2, 4<br>1 to 4<br>1	
Vukugah et al (2019)	NS	NA	NA
Naz et al (2021)	NS	NA	NA
Pirmahmadzoda et al (2021)	NS	NA	NA
Schaaf et al (2020)	NS	NA	NA
Kalawadia et al (2024)	Behavioural problems (4, 6.7%)<br>Subclinical sensorineural hearing loss (4, 6.7%)<br>Peripheral neuropathy (4, 6.7%)<br>Subclinical hypothyroidism (3, 5%)<br>Hypoalbuminemia (1, 1.7%)<br>Rashes (1, 1.7%)	NA	NS
Sharma et al (2020)	Gastric irritation (9, 75%)<br>Drug induced liver injury (4, 33%)<br>Hearing loss (2, 16.7%)<br>Anemia (6, 50%)<br>Hypothyroidism (1, 8.3%)<br>Rashes (1, 8.3%)	Unclear<br>Unclear<br>1<br>3<br>Unclear<br>Unclear	H, R
Das et al (2020)	Hypocalcaemia (1)<br>Hypoalbuminemia (1)<br>Optic neuritis (1)<br>Behavioural problems (1)<br>Anemia (2)<br>Sepsis/infection (3)<br>Hypokalemia (1)<br>Hypomagnesaemia (1)	1<br>2<br>3<br>3<br>3<br>3<br>3<br>4	Z, Cm, Lzd, Cs, Eto,
Jantarabenjakul et al (2022)	NS	NA	NA
Sun et al (2023)	NS	NA	NA
Schäfer et al (2023)	Hearing loss<br>Vision impairment<br>Hypothyroidism<br>Neutropenia<br>Thrombocytopenia<br>Hepatitis<br>Nausea	Unclear	Lzd, Pto, Amk
Khantee et al (2021)	Hepatitis (9, 5.1%)<br>Nausea and/or vomiting (6, 3.4%)<br>Rashes (2, 1.13%)<br>Peripheral neuropathy (1, 0.6%)<br>Ototoxicity (1, 0.6%)<br>Hypothyroidism (1, 0.6%)<br>Eosinophilia (1, 0.6%)	Unclear	NS

Note: The data above is for studies excluded from the meta-analysis, but included to ensure extensive survey of current literature. *Number and proportion of XDR cases. †Personal communication with the clinical investigator (Seddon, James. Conversation with: Jay Patra. 2015 December 08, 17.). Tx: Treatment, DST: Drug sensitivity testing, CSF: Cerebrospinal fluid, N A: Not applicable, NS: Not specified, GI: Gastrointestinal, H: isoniazid, R: rifampicin, EFV: efavirenz, D4T: stavudine, Ct: Colistin, Z: Pyrazinamide, Rfb: rifabutin, Km: kanamycin, Amk: amikacin, Cm: capreomycin, Cfx: Ciprofloxacin, Gfx: Gatifloxacin, Eto: ethionamide, Pto: protionamide, Cs: Cycloserine, Trd: terizidone, PAS: p-aminosalicylic acid, Cfz: clofazimine, Lzd: linezolid.
