# Supplementary material for: Treatment outcomes among children and adolescents with extensively drug–resistant (XDR) and pre–XDR tuberculosis: Systematic review and meta–analysis
Source: PLOS Glob Public Health. 2025 Jan 29;5(1):e0003754. doi: 10.1371/journal.pgph.0003754 (PMC11778756; doi:10.1371/journal.pgph.0003754)
Supplement: S6 Table — Note: FQ: Fluroquinolone, DST: Drug susceptibility testing, MDR: Multi–drug resistant, XDR: Extensively drug resistant, ref: Reference category. (PDF) [file pgph.0003754.s006.pdf]

S6 Table: Pooled treatment success among various subgroups of patients

Sub–groups	Treatment success (95% CI)	Heterogeneity (I^2)	Meta–regression	
			coeff (95%)	p–value
**DST confirmatory testing**				
≤50% of sample	100 (61–100)	..	-0·58 (-12·7–11·6)	0·916
>=50% of sample	75·8 (57·2–91·2)	46·90%	ref	
**Culture positive for XDR TB**				
≤50% of sample	100 (61–100)	..	-0·58 (-12·7–11·6)	0·916
>50% of sample	75·8 (57·2–91·2)	46·90%	ref	
**Treatment duration**				
≤18 mos	76·1 (48·5–96·5)	66·10%	-0.47(-7.7–6.8)	0·886
>18 mos	86·4 (67·2–99)	0·00%	ref	
**Use of injectable drugs**				
Yes	80·6 (58·8–96·7)	38·20%	5·5 (2·2–8·7	0·004
No	..	..	ref	
**Use of FQ**				
>=50% of sample	76·6 (52·7–95·1)	38·70%	0·59 (-13·4–14·6)	0·921
No	100 (51–100)	..	ref	
**Median age**				
≤5 years	78·6 (41–100)	78·40%	4·66 (-3·6–12·9)	
>5 years	80·1 (51·7–99)	50%	ref	0·223
**HIV Status**				
Positive	62·2 (25·8–93·3)	0%	-2·5 (-14·7–9·6)	0·617
Negative	86·6 (67·8–99)	0%	ref	
**Contact with an MDR or XDR TB patient**				
Yes	75 (26·3–100)	69·60%	-7·0(-18·2–4·2)	0·156
No	89·9 (71·3–100)	0%	ref	

Note: FQ: fluroquinolone, DST: drug sensitivity testing, MDR: Multi-drug resistant, XDR: Extensively drug resistant, ref: Reference category.
