# Supplementary material for: Treatment outcomes among children and adolescents with extensively drug–resistant (XDR) and pre–XDR tuberculosis: Systematic review and meta–analysis
Source: PLOS Glob Public Health. 2025 Jan 29;5(1):e0003754. doi: 10.1371/journal.pgph.0003754 (PMC11778756; doi:10.1371/journal.pgph.0003754)
Supplement: S7 Table — (PDF) [file pgph.0003754.s007.pdf]

S7 Table: Treatment percent (%) success, failure/default, and death.

Authors	Success (%)	Success 95% CI L	Success 95% CI U	N	Failure or Default (%)	Death (%)
Individualized treatment						
Schaaf et al. (2003)	53.9	38.6	68.4	100	21	…
Palacios et al. (2009)	60.5	44.7	74.4	85	18	13
Oliveira et al. (2011)	40	11.8	76.9	150	14	14
Satti et al. (2012)	79	56.7	91.5	120	…	12
Gegia et al. (2013)	60	45.5	73	200	20	3
Isaakidis et al. (2013)	9	1.6	37.7	50	27	64
Mignone et al. (2013)	95.5	78.2	99.2	80	5	…
Shin et al. (2003)	71.4	35.9	91.8	100	28	14
Marks et al. (2014)	77	69.3	83.3	140	2	9
Standardized treatment						
Mukherjee et al. (2003)	87.5	64	96.5	90	13	…
Thomas et al. (2010)	99	51	100	20	…	0
Fairlie et al. (2011)	53.8	29.2	76.8	130	16	31
Tabarsi et al. (2011)	99	56.6	100	15	0	0
Torres et al. (2011)	86.7	62.1	96.3	110	…	13
Seddon et al. (2012)	82	73.8	88	95	7	10
Seddon et al. (2012)	45.5	21.3	72	160	…	14
Anderson et al (2013)	70.6	64	76.4	105	19.6	6.9
Seddon et al. (2013)	67.8	60	74.8	150	6	2
Williams et al. (2013)	70.6	46.9	86.7	110	…	6
Aung et al. (2014)	91.3	84.7	95.2	75	9	…
Chan et al. (2004)	75.1	68.8	80.5	102	…	12
Hicks et al. (2014)	78.6	68.7	86	130	11	11
