# Supplementary material for: Treatment outcomes among children and adolescents with extensively drug–resistant (XDR) and pre–XDR tuberculosis: Systematic review and meta–analysis
Source: PLOS Glob Public Health. 2025 Jan 29;5(1):e0003754. doi: 10.1371/journal.pgph.0003754 (PMC11778756; doi:10.1371/journal.pgph.0003754)
Supplement: S2 Text — (PDF) [file pgph.0003754.s010.pdf]

S1 Protocol Study protocol for systematic review and meta-analysis to determine the clinical treatment outcomes of extensively drug-resistant (XDR) and pre-XDR tuberculosis (TB)*

Patra et al. **"Treatment outcomes among children and adolescents with extensively drug-resistant (XDR) and pre-XDR tuberculosis: systematic review and meta-analysis"**

Objective

In this systematic review and meta-analysis, we aim to investigate the clinical outcomes of XDR- and pre-XDR-TB in children and adolescents, as well as to identify challenges associated with treatment.

Inclusion criteria

Study type

- All study types will be eligible to enter meta-analysis.
- We will include all studies which were published as original report and present information on the relation between XDR- and pre-XDR-TB and clinical treatment outcomes.

Participants

- Children and adolescents, under 18 years of age will be included.
- If data for treatment outcomes for different age groups were reported, we will extract data for ages under 18 years.
- We will perform sub-group analyses through stratification by: DST confirmatory testing, culture positivity, use of injectable drugs and use of fluoroquinolone (each ≤50% or >50% of the cohort); treatment duration (≤ 18 months or >18 months); median age (≤ 5 years or >5 years); HIV status (positive or negative in the cohort); and contact with a MDR/XDR TB patient (yes or no).

Definition of exposition

- All studies which reported information on the treatment of XDR- or pre-XDR-TB in children and adolescents less than 18 years of age will be included.

Outcome variable

- All studies which reported treatment outcomes for XDR- and/or pre-XDR-TB in at least one age (less than 18 years of age) will be included
- Treatment outcomes, such as treatment success (cure, treatment completion) and treatment categories (death, failure, adverse events) will be stratified

Publication type

- Full published papers will be eligible (no language restrictions), including case studies.

Search Methods

We will search the following electronic databases:

- MEDLINE (via PubMed)
- EMBASE
- Scopus
- Web of Science
- Google Scholar
- Trial registries

There will be no restriction on language or year of publication. In these databases, we will search according to the thesaurus of the NCBI MESH browser the following terms and combinations of keywords in full text:

The following keywords will be employed:

1. ''tuberculosis''
2. "multidrug resistance" OR "multidrug-resistant" OR "extensively drug-resistant"
3. "XDR" OR "Pre-XDR"
4. "treatment outcomes"
5. "children" OR "adolescents"
6. "1" AND "4" AND "5" AND ("2" OR "3")

Additionally, bibliographies of identified publications and published reviews, online archives from the *International Journal of Tuberculosis and Lung Disease,* and conference abstracts from the *International Union Against Tuberculosis and Lung Disease (2004-15)* will be hand searched for potentially relevant articles. Authors will be contacted if data, methods and/or parameter definitions provided from the respective studies are unclear.

Reviews

All references cited in the identified reviews will be manually searched for potentially relevant studies.

Data collection

Two reviewers (JL, MB) will independently scrutinize the list of titles, and if available the abstracts, to determine potential usefulness of the article. Final selection will be based on the full text of potentially relevant articles by the two reviewers independently. In cases of disagreement, a third reviewer (JP) will examine such articles. Results will be discussed until

reaching consensus among all three reviewers. Study quality will be measured using the modified Newcastle–Ottawa scale (Wells et al. 2011).

The following study and patient characteristics will be extracted: setting, age, gender, method of diagnosis, HIV status and comorbid conditions, previous treatment and contact history, and the study outcome definitions. Treatment regimen characteristics will also be extracted, including: drugs used and DST results, duration of treatment, and length of follow-up. To assess study quality, information on variables that could have affected treatment success will be extracted: DST-based individualized treatment, use of injectable drugs and/or fluoroquinolones admission to hospital at the initiation of treatment and direct observation throughout the treatment. From all eligible studies, relevant data will be abstracted in duplicate, using a standardized data extraction sheet. An independent reviewer will confirm all data entries and will check at least twice for completeness and accuracy.

Meta-analysis & Meta-regression

Study Synthesis

- Data on numbers of subjects with and without clinical outcomes through treatment of XDR- and pre-XDR-TB and corresponding proportions and 95% credible intervals (95% CI) will be calculated.
- DerSimonian and Laird random-effects AND Bayesian random-effects models will be used to estimate pooled proportions for each XDR- and pre-XDR-TB outcomes.

Assessment of heterogeneity

- Impact of heterogeneity will be assessed by calculating the I^2 according to Higgins et al (Higgens JP et al 2003)
- Overall variation attributable to between study heterogeneity will be assessed by calculating the τ^2 statistic according to Rücker et al (Rücker G et al 2008)

Influence analysis

- Robustness of the pooled estimates will be checked by influence analyses. Each of the studies will be individually omitted from the data set, followed in each case by recalculation of the pooled estimate of the remaining studies.

Sensitivity/subgroup analyses

- As a sensitivity analysis, we will report pooled proportions with 95% credible intervals on all outcomes using a Bayesian random-effects model with Monte Carlo Markov chain simulations with non-informative priors and Gibbs sampling for the mean and variance parameters.

- Further stratifications will be made by DST confirmatory testing (≤50% or >50% of the cohort), treatment duration (≤ 18 months or >18 months), use of injectable drugs (≤80% or >80% of the cohort), age (≤ 5 years or >5 years), and HIV status (≤30% or >30% of the cohort)
- Meta-regression will be used to evaluate whether effect size estimates are significantly different by specific study characteristics and quality factors. Meta-regression coefficients and p-values will be provided.
- Forest plots will be plotted with summary points.

Evaluation of bias and confounding

Publication bias

- Publication bias will be assessed by inspection of the funnel plot and formal testing for funnel plot asymmetry, using Begg's test (Sterne JA et al. 2001).

Discussion and Evaluating

- The results will be critically and integratively discussed.

References

Higgins JP, Thompson SG, Deeks JJ, Altman DG (2003) Measuring inconsistency in meta-analysis. BMJ 327: 557-560.

Rücker G, Schwarzer G, Carpenter JR, Schumacher M. Undue reliance on I2 in assessing heterogeneity may mislead. BMC medical research methodology. 2008;8(1):79.

Sterne JA, Egger M, Smith GD (2001) Systematic review in health care: investigating and dealing with publication and other biases in meta-analysis. BMJ 323: 101-105.

Wells, G. A., Shea, B., O'Connell, D., Peterson, J., Welch, V., et al. The Newcastle-Ottawa Scale (NOS) for assessing the quality of nonrandomized studies in meta-analysis. 2011. http://www.ohri.ca/programs/clinical_epidemiology/oxford.asp
