# Supplementary material for: Treatment outcomes among children and adolescents with extensively drug–resistant (XDR) and pre–XDR tuberculosis: Systematic review and meta–analysis
Source: PLOS Glob Public Health. 2025 Jan 29;5(1):e0003754. doi: 10.1371/journal.pgph.0003754 (PMC11778756; doi:10.1371/journal.pgph.0003754)
Supplement: Attachment — Submitted filename: Response to Reviewers.pdf [file pgph.0003754.s013.pdf]

Response to Reviewer Comments

Thank you for the detailed and constructive feedback on our paper and we appreciate the opportunity to address the reviewer's comments which we believe have strengthened the manuscript. Below we have outlined responses to each comment individually and have submitted a clean and tracked changes version of the manuscript.

Reviewer #1: The systematic review estimated the pooled frequency of treatment outcomes (such as cure, treatment completion, death, and adverse events) among children with pre-XDR or XDR TB. The topic is very interesting and holds significant clinical importance. However, I have several major comments:

a. Publication Dates of Included Studies: All included studies were published before 2015. This means that novel drugs such as bedaquiline and delamanid were not used in these studies, and some new treatment options (such as BPaL) were also not considered. This inherent limitation may lead to an underestimation of the conclusions.

> **RESPONSE:** Based on our findings and systematic review, despite the increasing uptake of novel therapies such as delamanid and bedaquiline for the treatment of MDR-TB and XDR-TB, offering favourable outcomes in adults, there remains limited evidence at present to support its use in pediatric patients; hence, why our study did not consider the use of these recently approved drugs in our analysis

b. Sample Size and Treatment Complexity: The *sample sizes of each included study are too small*, which could affect the accuracy of the findings. Additionally, the complexity of treatment choices should not be ignored, as it significantly impacts the assessment of treatment outcomes.

> **RESPONSE:** We recognize that the sample sizes of the included studies in our paper are relatively small, especially since a number of case series were included. However, considering the challenges of diagnosing pediatric XDR–TB, the high-cost associated with treatment complexity, limited availability of second-line pediatric-approved drugs, and the prolific incidence of adverse events reported in adults, a lack of large–scale studies in this population is anticipated; hence, a limitation and gap in literature also highlighted through our paper.
>
> Methodologically, to tackle this issue a Bayesian approach was in fact adopted that deals with data with smaller samples without losing powers while retaining precision (Schoot et al 2014; 2015). Some citations are attached below FYI that we have incorporated in our study strength and limitation section.

Rens van de Schoot, Joris J. Broere, Koen H. Perryck, Mariëlle Zondervan-Zwijnenburg, and Nancy E. van Loey. Analyzing small data sets using Bayesian estimation: the case of posttraumatic stress symptoms following mechanical ventilation in burn survivors, Eur J Psychotraumatol. 2015; 6: 10.3402/ejpt.v6.25216. Published online 2015 Mar 11. doi: 10.3402/ejpt.v6.25216

Van de Schoot R, Kluytmans A, Tummers L, Lugtig P, Hox J, Muthén B. Facing off with Scylla and Charybdis: A comparison of scalar, partial, and the novel possibility of approximate measurement invariance. Frontiers in Psychology. 2013;4:770.

c. Revised Definitions of Pre-XDR and XDR TB: *The definitions of pre-XDR and XDR TB were revised in 2021*. This limits the value of the conclusions, as they cannot be generalized to the current situation.

RESPONSE: We appreciate this insightful comment and have revised our manuscript to explicitly state this limitation. The revision of the defining diagnostic criteria for pre-XDR TB and XDR TB by the WHO in 2021 may indeed contribute to inconsistencies in the findings reported across studies. This classification bias potentially impacts the reliability of the reported effect sizes and contributes to the observed statistical heterogeneity, even though it remains relatively low. This recognition is crucial for accurately interpreting our conclusions within the context of evolving diagnostic standards.

Reviewer #2: 1. The introduction provides a good overview of the prevalence and significance of XDR and pre-XDR tuberculosis. However, it could be strengthened by adding more detail on the specific challenges of diagnosing and treating these conditions in pediatric populations.

RESPONSE: Thank you for your feedback. We have taken your suggestion into consideration and have made the necessary revisions in the introduction. We have expanded our introduction section to explicitly address these points.

2. Why no mention about publication bias based on funnel plot or doi plot. Kindly do grade analysis to give a more reliable met analysis.

RESPONSE: Thank you for your feedback and suggestions regarding the assessment of publication bias and the use of GRADE analysis. We understand the importance of evaluating publication bias to ensure the robustness of our meta-analysis. In response to your comment, we have now included a funnel plot based on the study success rates using proportion data, as outlined in the attached figures. For the outcomes of death and failure, we did not initially have data on the denominators. Therefore, we calculated the sample sizes using the confidence intervals (CI) of the success proportion and generated funnel plots for each outcome, except for adverse events where the data were too sparse to perform a meaningful analysis. Funnel plots have also been provided for study

death rates and failure/default rates. We believe these additional analyses provide a more comprehensive assessment of potential publication bias in our study and strengthen the reliability of our findings. Thank you again for your valuable feedback. We have updated the limitations section of the manuscript to reflect these additions and added all 3 funnel plots as part of Supplementary Figure 1.

**Authors**	**Success (%)**	**Success 95% CI L**	**Success 95% CI U**	**N**	**Failure or Default (%)**	**Death (%)**
Individualized treatment						
Schaaf et al. (2003)	53.9	38.6	68.4	100	21	…
Palacios et al. (2009)	60.5	44.7	74.4	85	18	13
Oliveira et al. (2011)	40	11.8	76.9	150	14	14
Satti et al. (2012)	79	56.7	91.5	120	…	12
Gegia et al. (2013)	60	45.5	73	200	20	3
Isaakidis et al. (2013)	9	1.6	37.7	50	27	64
Mignone et al. (2013)	95.5	78.2	99.2	80	5	…
Shin et al. (2003)	71.4	35.9	91.8	100	28	14
Marks et al. (2014)	77	69.3	83.3	140	2	9
Standardized treatment						
Mukherjee et al. (2003)	87.5	64	96.5	90	13	…
Thomas et al. (2010)	99	51	100	20	…	0
Fairlie et al. (2011)	53.8	29.2	76.8	130	16	31

Tabarsi et al. (2011)	99	56.6	100	15	0	0
Torres et al. (2011)	86.7	62.1	96.3	110	…	13
Seddon et al. (2012)	82	73.8	88	95	7	10
Seddon et al. (2012)	45.5	21.3	72	160	…	14
Anderson et al (2013)	70.6	64	76.4	105	19.6	6.9
Seddon et al. (2013)	67.8	60	74.8	150	6	2
Williams et al. (2013)	70.6	46.9	86.7	110	…	6
Aung et al. (2014)	91.3	84.7	95.2	75	9	…
Chan et al. (2004)	75.1	68.8	80.5	102	…	12
Hicks et al. (2014)	78.6	68.7	86	130	11	11

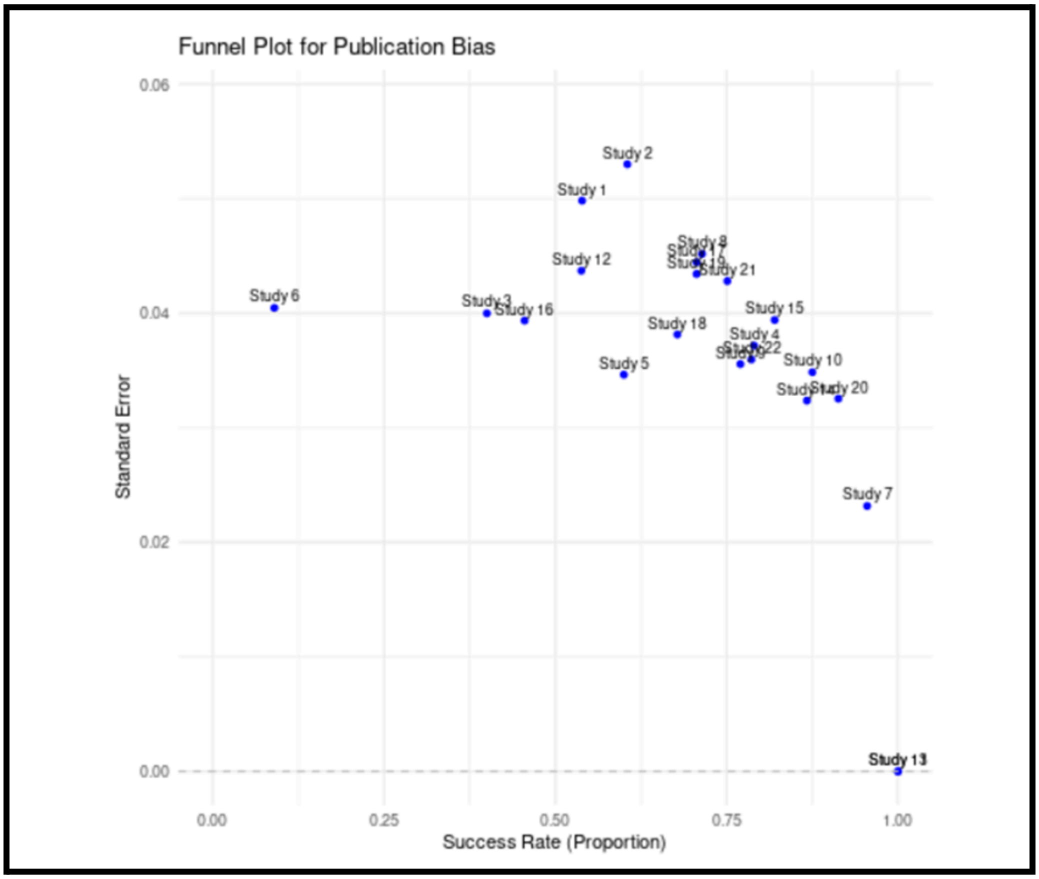

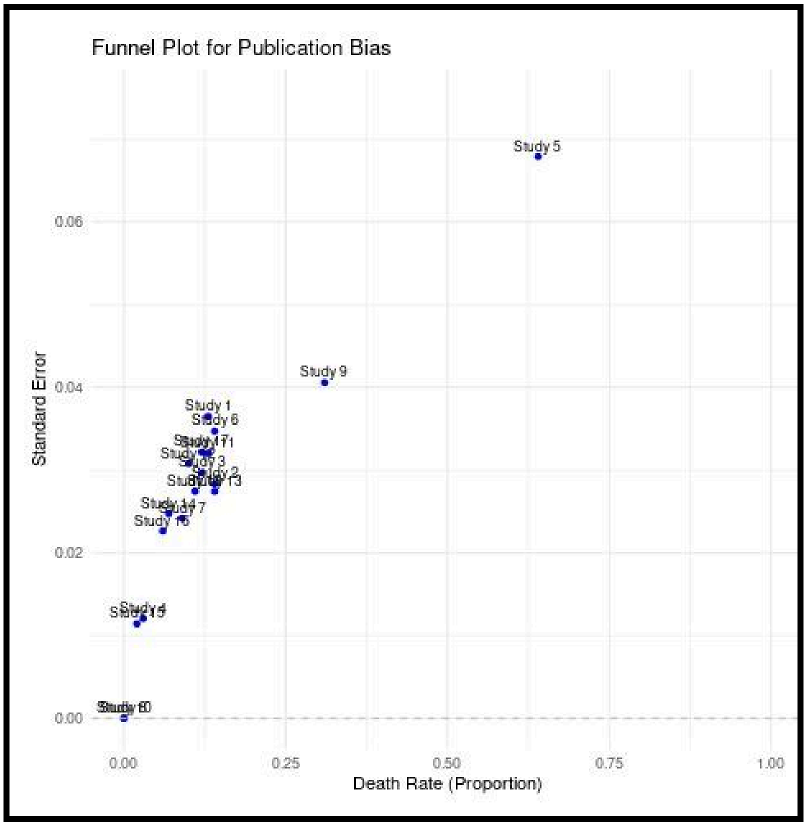

Funnel Plot for Publication Bias
Standard Error
Death Rate (Proportion)
0.00
0.02
0.04
0.06
0.00
0.25
0.50
0.75
1.00
Study 5
Study 9
Study 1
Study 6
Study 7
Study 14

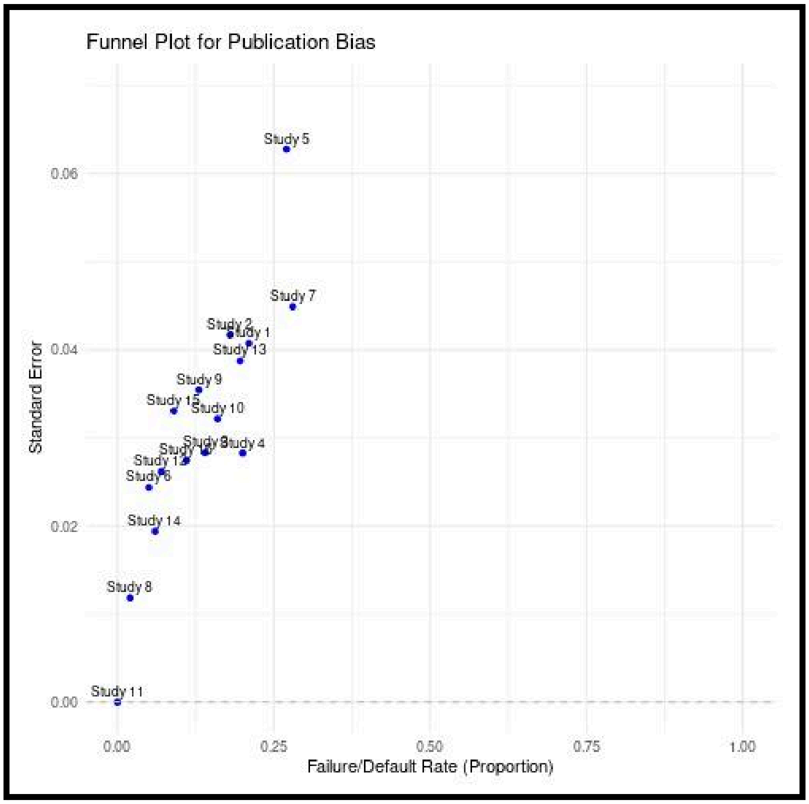

Funnel Plot for Publication Bias
Standard Error
Failure/Default Rate (Proportion)
0.00
0.02
0.04
0.06
0.00
0.25
0.50
0.75
1.00
Study 5
Study 7
Study 13
Study 9
Study 10
Study 4
Study 6
Study 14
Study 8
Study 11

3. *Sensitivity analysis* component is also missing as a part of meta analysis.

> **RESPONSE:** We conducted sensitivity analyses using stratified analysis, which can be found in Supplementary Table 2. However, due to the limited number of data points available for some outcomes, we were unable to perform a meta-regression analysis for outcomes other than the success rate. As a result, our sensitivity (subgroup) analyses were primarily focused on and limited to success rate to ensure the robustness of our findings. We appreciate your feedback and have clarified this in the revised manuscript to reflect the limitations and the scope of our sensitivity analyses.

4. The reporting of adverse events is crucial. It would be helpful to categorize these adverse events by severity to better understand the treatment burden.

> **RESPONSE:** Thank you for your feedback. To clarify, the severity of adverse events detailed in Supplementary Table 6 is described in terms of grade between the range of 1 to 4 with 1 being indicative of low severity and 4 being indicative of high severity. This categorization provides a clearer understanding of the treatment burden associated with each adverse event.

Reviewer #3:

Introduction:

1. The introduction could be expanded to provide further insights into the burden of TB among children and adolescents. This section could detail the proportion of TB cases in children that are pulmonary versus extrapulmonary, as well as the breakdown of drug-sensitive TB cases. Additionally, it could include statistics on drug-resistant TB, specifying the incidence of multidrug-resistant (MDR), pre-extensively drug-resistant (pre-XDR), and extensively drug-resistant (XDR) TB

> **RESPONSE:** Thank you for your feedback. We have taken your suggestion into consideration and have made the necessary revisions in the introduction. We have expanded our introduction section to explicitly address these points.

2. The section on treatment benefits from a more comprehensive overview of the various treatment options currently practiced worldwide. This could include standard treatment protocols, variations in treatment strategies across different regions, and the latest advancements in TB treatment.

> **RESPONSE:** Thank you for your feedback. We have taken your suggestion into consideration and have made the necessary revisions in the introduction. We have expanded our introduction section to explicitly address these points.

Methodology:

1. List the *specific inclusion and exclusion criteria used* for selecting studies like extrapulmonary TB cases included or excluded.

> **RESPONSE:** We included all types of TB cases, including both pulmonary and extrapulmonary TB, as specified in the full study protocol for systematic review and meta-analysis detailed in Supplementary Text 2. This comprehensive approach ensures that our analysis encompasses the full spectrum of TB presentations, thereby enhancing the robustness and generalizability of our findings.

2. Details on the *data extraction* process – any *specific template used* may be mentioned for reproducibility

> **RESPONSE:** All study and patient-related characteristics were extracted using the data extraction tool developed on excel (refer to Appendix I).

3. *Describe how missing data were handled* in the analysis, particularly for key outcomes.

> **RESPONSE:** Thank you for your comment regarding the handling of missing data in our analysis. We acknowledge the importance of addressing missing data, particularly for our 3 key outcomes. In our study, missing data were present, but they were limited in scope. Specifically, only a few studies had missing data for the initial three outcomes: success (5 studies), death (5 studies), and failure (3 studies). In contrast, a more substantial amount of missing data was observed for adverse events. To address this, we provided a detailed description of the reported adverse events in the included studies in Supplementary Table 6.

4. The R^2 *value may be reported for Brooks–Gelman criteria* to understand the model convergence in Monte Carlo Markov chain simulations models.

> **RESPONSE:** In this meta-analytic example, we aimed to estimate the pooled frequency of treatment outcomes (such as cure, treatment completion, death, and adverse events) among children with pre-XDR or XDR TB. We began by calculating the log-relative risk for each study, a common method for meta-analyzing binary outcomes (Higgins et al., 2021). Next, we selected our parameters, choosing a random-effects model because we anticipated that each study would capture a slightly different, though related, true effect size based on the population studied. Since we didn't have any predetermined covariates of interest that were consistent across the studies, we did not include additional parameters as would be done in a meta-regression. Our model, therefore, included two key parameters to estimate: the model intercept (a fixed effect) and tau (a random effect). We also re-estimated the effect sizes for each study as random effects. Alternatively, instead of re-estimating the study effect sizes, we

could have opted to estimate an additional variance parameter alongside tau, resulting in a model with three parameters—the intercept, tau, and a new variance parameter (Nicenboim et al., 2021). After estimation, tau provided a measure of variability in effect sizes across studies, while the model intercept represented our pooled effect size on the log-relative risk scale. Therefore, reporting r^2 was redundant.

Results:

1. Results of sub-group analysis particularly on different treatment regiments and its effect on treatment outcomes can add more value for the study and address the interest of the readers

RESPONSE: Thank you for your comment regarding the inclusion of subgroup analyses on different treatment regimens and their effects on treatment outcomes. We appreciate the suggestion and agree that such analyses could provide valuable insights. However, due to the limited number of studies included in our meta-analysis, we did not have enough data points to conduct meta-regression analyses for outcomes other than the success rate. As a result, our sensitivity (subgroup) analyses were restricted to examining the success rate alone. We recognize that exploring treatment regimens in greater detail could offer additional value, and we have noted this limitation in the revised manuscript. Future research with a larger dataset could provide the opportunity to conduct more comprehensive subgroup analyses and better address the impact of different treatment regimens on various outcomes.

OUTCOME ANALYSIS							
				TOTAL (XDR + PRE-XDR)			
		denom	success	failordef	death	adverse	remainDK
TOTAL		81	64	10	9	23	7

Discussion:

1. Discuss the findings in a broader context like how they align with or challenge current TB treatment guidelines and policies for children and adolescents

RESPONSE: Thank you. We have updated the manuscript accordingly.

Limitations:

1. Elaborate on specific limitations related to study designs, such as retrospective versus prospective studies, and the impact of varying definitions and diagnostic criteria across studies.

RESPONSE: We have expanded our discussion section to explicitly address these points. We agree that the inclusion of both prospective and retrospective studies poses another key limitation as both study designs are susceptible to

their own unique biases. On one hand, retrospective studies rely primarily on historical self-reported data by participants, physicians, and/or researchers, and are prone to selection and recall bias; on the other hand, prospective studies, while generally more robust, may still be subject to temporal bias owing to their time-dependent confounding variables.

2. Discuss the potential impact of publication bias and describe any statistical tests (e.g., Egger's test) used to assess it.

RESPONSE: Thank you for pointing out this and we have incorporated your suggestion and have added funnel plots as supplementary materials (Supplementary Figure 1 – Panel A,B,C).

Recommendations:

1. Provide recommendations for clinical practice based on the study findings, such as potential adjustments to treatment regimens or monitoring protocols for this age group.

RESPONSE: Thank you for your feedback! The manuscript has been updated accordingly.
