# Supplementary material for: Treatment outcomes among children and adolescents with extensively drug–resistant (XDR) and pre–XDR tuberculosis: Systematic review and meta–analysis
Source: PLOS Glob Public Health. 2025 Jan 29;5(1):e0003754. doi: 10.1371/journal.pgph.0003754 (PMC11778756; doi:10.1371/journal.pgph.0003754)
Supplement: Attachment — Submitted filename: renamed_2b292.docx [file pgph.0003754.s012.docx]

**Review of “Treatment outcomes among children and adolescents with extensively drug–resistant (XDR) and pre–XDR tuberculosis: systematic review and meta–analysis”**

**Introduction:**

1. The introduction could be expanded to provide further insights into the burden of TB among children and adolescents. This section could detail the proportion of TB cases in children that are pulmonary versus extrapulmonary, as well as the breakdown of drug-sensitive TB cases. Additionally, it could include statistics on drug-resistant TB, specifying the incidence of multidrug-resistant (MDR), pre-extensively drug-resistant (pre-XDR), and extensively drug-resistant (XDR) TB
2. The section on treatment benefits from a more comprehensive overview of the various treatment options currently practiced worldwide. This could include standard treatment protocols, variations in treatment strategies across different regions, and the latest advancements in TB treatment.

**Methodology:**

1. List the specific inclusion and exclusion criteria used for selecting studies like extrapulmonary TB cases included or excluded.
2. Details on the data extraction process – any specific template used may be mentioned for reproducibility
3. Describe how missing data were handled in the analysis, particularly for key outcomes.
4. The R^ value may be reported for Brooks–Gelman criteria to understand the model convergence in Monte Carlo Markov chain simulations models.

**Results:**

1. Results of sub-group analysis particularly on different treatment regiments and its effect on treatment outcomes can add more value for the study and address the interest of the readers

**Discussion:**

1. Discuss the findings in a broader context like how they align with or challenge current TB treatment guidelines and policies for children and adolescents

**Limitations:**

1. Elaborate on specific limitations related to study designs, such as retrospective versus prospective studies, and the impact of varying definitions and diagnostic criteria across studies.
2. Discuss the potential impact of publication bias and describe any statistical tests (e.g., Egger's test) used to assess it.

**Recommendations:**

1. Provide recommendations for clinical practice based on the study findings, such as potential adjustments to treatment regimens or monitoring protocols for this age group.
